# Endothelial Function in Postmenopausal Women: The Possible Role of Heat Shock Protein 60 and Serum Androgens

**DOI:** 10.3389/fmmed.2022.933188

**Published:** 2022-07-12

**Authors:** Eleni Armeni, Anastasia Soureti, Areti Augoulea, Asimina Chondrou, Nikolaos Drakoulis, George Kaparos, Dimitrios Delialis, Spyros Stefos, Lasthenis Angelidakis, Alexandros Sianis, Aggeliki-Maria Dimopoulou, Andreas Alexandrou, Stavroula Baka, Leon Aravantinos, Konstantinos Panoulis, Kimon Stamatelopoulos, Irene Lambrinoudaki

**Affiliations:** ^1^ 2^nd^Department of Obstetrics and Gynecology, National and Kapodistrian University of Athens, Aretaieio Hospital, Athens, Greece; ^2^ Research Group of Clinical Pharmacology and Pharmacogenomics, Faculty of Pharmacy, School of Health Sciences, National and Kapodistrian University of Athens, Athens, Greece; ^3^ Hormonal and Biochemical Laboratory, 2^nd^Department of Obstetrics and Gynecology, National and Kapodistrian University of Athens, Aretaieio Hospital, Athens, Greece; ^4^ Laboratory of Therapeutics and Vascular Pathophysiology, National and Kapodistrian University of Athens, Alexandra Hospital, Athens, Greece

**Keywords:** heat shock protein 60, flow mediated dilation, testosterone, dehydroepiandrosterone, postmenopause

## Abstract

**Background:** Heat shock protein 60 (HSP60), a potentially homeostatic antigen, is involved in physiological and non-physiological conditions. Experimental data support the role of HSP60 in placental and mitochondrial steroidogenesis. Furthermore, HSP60 is translocated into the endothelial-cell plasma membrane and the extracellular space under stress conditions, promoting the atherosclerotic process. Therefore, we investigated the association between HSP60 and endothelial function in postmenopausal women, considering the possible atherogenic effect of androgenic hormones.

**Methods:** This study included 123 healthy postmenopausal women. Exclusion criteria were treated hypertension or dyslipidaemia, menopause hormone therapy during the last 6 months, and previously diagnosed peripheral vascular disease or cardiovascular disease. Fasting venous blood samples were obtained for biochemical and hormonal assessment and evaluation of HSP60. Sonographic assessment of flow-mediated dilation (FMD) occurred immediately after that in one session.

**Results:** Univariate analysis showed that women with FMD values below median 5.12% had lower logHSP60 values (low vs. high FMD, HSP60 values: 2.01 ± 1.16 ng/ml vs. 3.22 ± 1.17 ng/ml, *p*-value = 0.031). Multivariable analysis showed that logHSP60 was associated with FMD (b-coefficient = 0.171, *p*-value = 0.046), adjusting for traditional cardiovascular risk factors (TRFs) and insulin levels. Further adjustment for testosterone and DHEAS rendered the result non-significant. In the multivariable analysis, FMD was associated with insulin (b-coefficient = −0.166, *p*-value = 0.034), testosterone (b-coefficient = −0.165, *p*-value = 0.034), DHEAS (b-coefficient = −0.187, *p*-value = 0.017), adjusting for TRFs.

**Discussion:** The results of this study indicate that the association between androgens and endothelial function is possibly mediated by HSP60 molecules, in women with low insulin resistance and androgenicity. Further prospective studies are needed to explore the significance of our findings.

## Introduction

Heat shock proteins (HSP)s 60 have been considered antigens of particular immunoregulatory interest in cardiovascular homeostasis (Coelho and Faria, 2012; Duan et al., 2020). They have been shown to modify endothelial cell function, cardiomyocytes and vascular smooth muscle cell activity in both health and disease (Duan et al., 2020). An increasing number of diseases, known as chaperonopathies, are linked with qualitative or quantitative or functional changes of molecular chaperones, either in excess or defect or express an incorrect function (Cappello et al., 2014).

A large body of evidence supports a direct link between HSP60 and the function of the cardiovascular system. The intracellular HSP60 in cardiomyocytes has a protective role in maintaining mitochondrial integrity and triphosphate adenosine (ATP) capacity, which is essential for survival and regulation of reperfusion injury (Duan et al., 2020). In the event of a disruption of the mitochondrial chain integrity, HSP60 expression is upregulated to control the production of H_2_O_2_ and cytochrome c release (Hollander et al., 2003). In endothelial cells, HSP60 molecules seem to be translocated to the plasma membrane and the extracellular space under stress conditions (Duan et al., 2020). Higher levels of extracellular HSP60 have been shown to induce the proliferation and migration of vascular smooth muscle cells, further supporting the atherosclerotic process (Zhao et al., 2015; Deniset et al., 2018). Earlier data from *in vitro* studies indicated that extracellular HSP60 might promote the synthesis of proteolytic enzymes and cytokines like tumour necrosis factor (TNF), growth factors, interleukin-10 and adhesion molecules (Moghimpour Bijani et al., 2012).

HSP60 are molecules involved in various aspects of human reproduction, like modulation of endometrial steroid function (Neuer et al., 2000). *In vitro* evidence supports a direct involvement of HSP60 with human placental steroidogenesis (Olvera-Sanchez et al., 2011) and mitochondrial progesterone synthesis (Monreal-Flores et al., 2017). As evident from *in vitro* data, maximum values of HSP60 are expressed following regular ovulation, ensuring endometrial receptivity for possible implantation, as well as during the pre-and peri-implantation stages of pregnancy (Neuer et al., 2000). HSP60 proteins are expressed in human follicular fluid (Neuer et al., 1997) in the granulosa of primary and secondary atretic ovarian follicles and more significant amounts in the theca interna of tertiary and cystic follicles (Garrido et al., 2001).

Endothelial dysfunction is primarily characterized by reduced nitric oxide availability and is one of the first vascular alterations observed in the sequel of atherosclerosis, preceding the structural vascular changes as well as the clinical manifestations of cardiovascular disease (Moreau et al., 2012; Thijssen et al., 2019). Flow-mediated dilation (FMD) is the gold-standard non-invasive ultrasound-based technique for assessing endothelial function (Thijssen et al., 2019) and has been previously associated with prognostic value for future CV events (Inaba et al., 2010; Xu et al., 2014; Thijssen et al., 2019). We have previously shown that circulating androgens are predictors of accelerated vascular ageing and blood pressure increase after the menopausal transition (Georgiopoulos et al., 2016). Therefore, we aimed to evaluate the possible association between HSP60 serum levels and endothelial function via FMD in a sample of apparently healthy postmenopausal women, considering the possible hormonal interactions.

## Materials and Methods

### Study Design and Population

This cross-sectional study evaluated healthy postmenopausal women examined in the Menopause Clinic of Aretaieio Hospital, 2nd Department of Obstetrics and Gynecology, National and Kapodistrian University of Athens. This clinic has been active since 1998, offering advice to all postmenopausal women seeking help on managing menopause-related symptoms and primary prevention practices. All women who visited the clinic for the first time and had a clinical frailty score of less than 5 (Rockwood et al., 2005) were invited to participate. The intake of vitamins or food supplements without phytoestrogens was not a criterion for exclusion.

Briefly, before their recruitment, all women were subjected to a routine evaluation program that included breast mammography, gynaecological examination and Papanicolaou smear, and evaluation of renal/thyroid/liver function. Exclusion criteria were: 1) familial hypercholesterolemia, 2) clinically overt or treated coronary artery disease or cardiovascular disease or family history of early atherosclerotic cardiovascular disease (1st-degree male relative < 55 years or 1st-degree female relative < 65 years), 3) thromboembolism as well as peripheral artery disease, 4) premature ovarian failure, gynaecological malignancy, 5) acute or chronic inflammatory disease, 6) use of antihypertensive, hypolipidemic medication or other vasoactive medications, 7) known diabetes mellitus or intake of related medication, 8) intake of hormone replacement therapy or selective estrogen modulators, 9) menopausal age of more than 10 years. Absence of menses for at least 12 consecutive months together with follicle-stimulating hormone > 25 mIU/ml and levels of estradiol < 50 pg/ml was defined as post-menopause. All participating women were only consuming alcohol under social circumstances (i.e., 2-3 glasses of wine per week). Women with adherence or retention concerns (e.g., alcoholism) were not included in the study. After applying the exclusion criteria, a total of 123 healthy women were selected to participate in this study. All participants signed informed consent for participation in the study. Institutional review board approval was obtained by the Ethics Committee of the “Aretaieio” Hospital.

### Biochemical and Hormonal Assays

Total cholesterol in serum was measured by enzymatic assay (Abbot, Illinois) with a total coefficient of variation ≤ 3% and sensitivity of 5.0 mg/dl. Triglycerides were assessed using the enzymatic glycerol phosphate oxidase methodology (Abbott), with a coefficient of variation ≤ 5% and a sensitivity of 5.0 mg/dl. The Ultra HDL assay (Abbott) was used to measure levels of High-density (HDL) lipoprotein cholesterol with a total coefficient of variation ≤ 4% and a sensitivity of 2.5 mg/dl. Low-density lipoprotein cholesterol (LDL-C) was measured by elimination methodology (MULTIGENT direct LDL, Abbott, Illinois). The sensitivity of the assay was ≤ 10 mg/dl, and the total coefficient of variation was <4%. Serum glucose was measured by the hexokinase/G-6-PDH methodology (Abbott). The total coefficient of variation was ≤ 5%, and the sensitivity was 2.5 mg/dl. All the assays as mentioned earlier were performed on the Architect c 8,000 system (Abbott Diagnostics, Illinois). HSP60 levels were measured using the Human Heat Shock Protein 60 ELISA kit (Cusabio Biotech, Newark, DE), with a sensitivity of 0.39 ng/ml. The intra- and inter-assay precision was reported as < 8% and <10%, respectively.

Levels of serum insulin, follicle-stimulating hormone (FSH), estradiol, sex hormone-binding globulin (SHBG), testosterone and dehydroepiandrosterone (DHEAS) were measured as previously described (Creatsa et al., 2012; Stamatelopoulos et al., 2015). In addition, homeostasis model assessment of insulin resistance (HOMA-IR) was calculated as follows: fasting insulin (μU/mL) x fasting glucose (mmol/L)/22.5.

### Flow-Mediated Dilation

FMD was assessed using high-resolution ultrasound (Vivid 7 Pro, G.E.) with a attached 7.0- to 14.0-Hz multifrequency linear array probe. The intraobserver CV was estimated as 8.2%. The right brachial artery was longitudinally imaged above the antecubital fossa in a supinated position of the forearm. The examiner placed a pneumatic cuff around the forearm, and the initial evaluation took place with the assessment of brachial diameter and flow velocity. The cuff was rapidly inflated to 250 mmHg for 5 min and subsequently deflated, causing an increase in the arterial flow (reactive hyperemia). After that, we monitored the extent of reactive hyperemia, velocity and changes in diameter for 90 s. FMD was calculated as the percentage of maximal change of lumen diameter between rest and reactive hyperemia (Stamatelopoulos et al., 2011, 2019). The interclass correlation coefficient for FMD measured on two successive mornings by the same investigator was 0.706.

### Statistical Analysis

Statistical analysis was performed by SPSS version 20.0 (SPSS, Chicago, IL, United States). Qualitative data are expressed as absolute counts and frequencies. Quantitative data are expressed as mean values and standard deviation (mean ± S.D.) or median (inter-quartile range). The normality of distributions was graphically inspected through histograms and Quantile-Quantile plots. Logarithmic transformation was used for non-normally distributed variables, i.e., HSP60 values. Values of HSP60 were compared with analysis of variance (ANOVA) and analysis of covariance (ANCOVA), using the median value of FMD as the cut-off. We performed correlation analysis by Pearson to evaluate links between markers of endothelial function and central blood pressure and any of the assessed molecules, as well as possible hormonal and metabolic determinants.

Moreover, we assessed possible links between values of HSP60 and serum levels of androgenic sex hormones and FSH. Subsequently, we fitted models of multivariable regression analysis, including vascular parameters as dependent variables (in a one-by-one fashion) and the assessed molecules and significant cardiovascular risk factors as independent determinants. Statistical significance was set at the level of *p-value* < 0.05.

## Results

**FIGURE 1:**
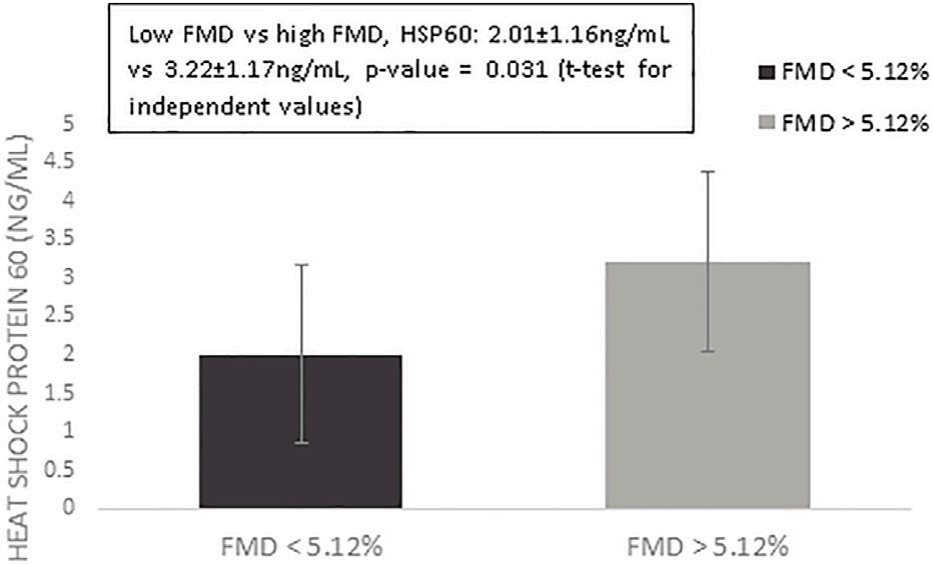
Mean values of log-transformed heat shock protein 60 for women with flow-mediated dilation above vs. below the median value of 5.12%. Error bars indicate standard errors.

Table 1 presents the results of the descriptive analysis for the n = 123 postmenopausal women in our study. We compared values of HSP60 and cardiovascular risk factors according to FMD measures, using the median as the cut-off. As presented in Figure 1, we observed that only HSP60 values differed significantly between women with low vs. high levels of FMD (FMD values < 5.12% vs. > 5.12%, comparison of HSP60 values: 2.01 ± 1.16 ng/ml vs. 3.22 ± 1.17 ng/ml, *p*-value = 0.031, t-test for independent values).

**TABLE 1:** Descriptive characteristics for the *n* = 123 postmenopausal women of our study.

	Mean ± SD or Frequency (%)	IQR
**Anthropometric/demographic parameters**		
Age (years)	55.5 ± 5.8	52.0–58.0
YSM (years)	7.58 ± 5.9	3.0–10.0
BMI (kg/m^2^)	25.9 ± 4.3	23.0–28.4
Overweight—obesity (%)	49.7% (79/159)	
SBP (mmHg)	119.3 ± 17.4	106.0–130.0
DBP (mmHg)	74.2 ± 9.1	67.8–81.0
Current smoking (%)	29.6% (47/159)	
**Biochemical parameters**		
Cholesterol (mg/dl)	230.4 ± 39.1	209.0–256.0
HDL-C (mg/dl)	62.0 ± 17.3	50.0–72.8
Triglycerides (mg/dl)	90.9 ± 39.9	66.0–102.0
LDL-C (mg/dl)	142.4 ± 36.7	114.5–168.0
Glucose (mg/dl)	92.1 ± 8.9	86.0–97.0
Insulin (μIU/ml)	7.1 ± 4.5	4.3–8.5
HOMA-IR	1.7 ± 1.2	0.9–1.9
C-reactive protein (mg/dl)	0.4 ± 0.7	0.1–0.3
**Hormonal parameters**		
FSH (mIU/ml)	76.4 ± 30.6	53.6–98.6
LH (mIU/ml)	37.7 ± 19.3	25.3–46.2
Testosterone (ng/dl)	0.4 ± 0.3	0.3–0.5
SHBG (nmol/L)	66.3 ± 28.4	45.1–86.3
FAI	2.5 ± 1.8	1.3–3.2
DHEAS (ng/dl)	345.2 ± 80.8	85.0–640.0
**Endothelial function**		
Flow mediated dilation (%)	5.4 ± 2.7	3.3–6.8

YSM, years since menopause; SBP, systolic blood pressure; DBP, diastolic blood pressure; HDL, high density lipoprotein; LDL, low density lipoprotein; HOMA-IR, homeostasis model assessment of insulin resistance; FSH, follicular stimulating hormone; LH, luteinizing hormone; SHBG, sex hormone binding globulin; FAI, free androgen index; SD, standard deviation; IQR, interquartile range.

We continued the analysis evaluating possible significant correlations between FMD values and hormonal, biochemical, anthropometric predictors as well as the levels of the assessed molecules (Table 2). The results indicated that FMD values correlated negatively with insulin (r-coefficient = −0.181, *p*-value = 0.046), DHEAS (r = −0.275, *p*-value = 0.013), testosterone (r-coefficient = -0.192, *p*-value = 0.028) and FAI levels (r-coefficient = −0.183, *p*-value = 0.046). We also observed an almost significant correlation between serum levels of HSP60 and FMD values (r-coefficient = 0.168, *p*-value = 0.069), as well as further almost significant correlations between FMD and HOMA-IR (r = −0.156, *p*-value0.087) or FSH (r = 0.197, *p*-value = 0.084). In addition, we evaluated the partial continuous correlation between values of HSP60 and the following parameters: DHEAS (r = 0.015, *p*-value = 0.908); testosterone (r = −0.233, *p*-value = 0.064); FSH (r = 0.255, *p*-value = 0.042) after adjustment for age, BMI, smoking and insulin levels.

**TABLE 2:** Correlation analysis between flow mediated dilation values and hormonal—metabolic predictors and heat shock protein 60.

	Flow mediated dilation	
	All women *n* = 123	
	*r-coefficient*	*p-value*
Age (years)	−0.098	0.221
YSM (years)	0.011	0.913
BMI (kg/m^2^)	−0.004	0.959
SBP (mmHg)	−0.075	0.350
DBP (mmHg)	−0.100	0.215
Cholesterol (mg/dl)	−0.049	0.558
HDL-C (mg/dl)	0.021	0.800
Triglycerides (mg/dl)	−0.054	0.515
LDL-C (mg/dl)	−0.021	0.803
Glucose (mg/dl)	0.041	0.625
Insulin (μIU/ml)	−**0.181**	**0.046**
HOMA-IR	−0.156	0.087
FSH (mIU/ml)	0.197	0.084
LH (mIU/ml)	0.056	0.501
DHEAS (ng/dl)	−**0.275**	**0.013**
Testosterone (ng/dl)	−**0.192**	**0.028**
SHBG (nmol/L)	0.083	0.357
FAI	−**0.183**	**0.046**
HSP60 (ng/ml)	0.168	0.069
C reactive protein (mg/dl)	−0.061	0.574

R-coefficient was derived from Pearson’s correlation Bold indicates statistical significance, which was at the level of *p*-value < 0.05.

YSM, years since menopause; BMI, body mass index; SBP, systolic blood pressure; DBP = diastolic blood pressure; HDL-C, high density lipoprotein cholesterol; LDL-Cl, low density lipoprotein cholesterol; HOMA-IR, homeostasis model assessment of insulin resistance; FSH, follicular stimulating hormone; LH, luteinizing hormone; SHBG, sex hormone binding globulin; FAI, free androgen index.

**FIGURE 2:**
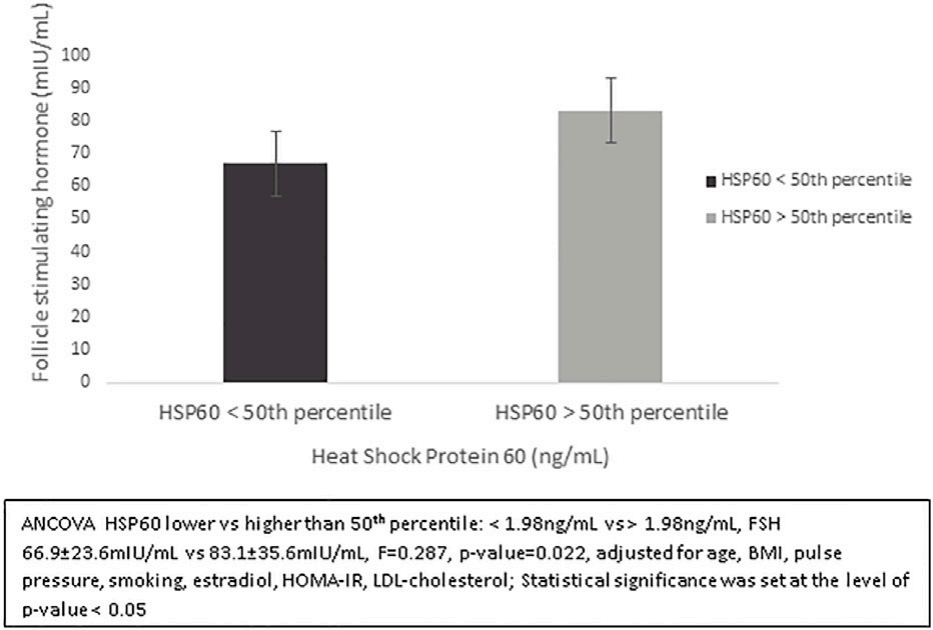
Follicle-stimulating hormones according to values of heat shock protein 60 (HSP60), stratified according to the median value of 1.98 ng/ml.

We evaluated the possible association between HSP60 values, stratified according to the median value and hormone levels. As shown in Figure 2, a significant association was observed only for FSH and HSP60 values, which persisted even after analysis for cardiovascular risk factors (<1.98 ng/ml vs. > 1.98 ng/ml, FSH: 66.9 ± 23.6 mIU/ml vs. 83.1 ± 35.6 mIU/ml, F = 0.287, *p*-value = 0.022, ANCOVA, adjusted for age, BMI, pulse pressure, smoking, estradiol, HOMA-IR, LDL-cholesterol). No other significant associations were observed between values of HSP60 and testosterone, insulin, HOMA-IR or DHEAS.

Table 3 presents the results of the multivariable linear regression analysis, including FMD values as dependent variable and HSP60 and significant confounders (i.e., age, pulse pressure, androgens, insulin). Model 1 shows that values of FMD associated significantly with HSP60 (b-coefficient = 0.171, 95% CI: 0.043 to 0.358, *p*-value = 0.046) in a model adjusted for age, pulse pressure, smoking, LDL-cholesterol, insulin and BMI. This association was not significant after further adjustment for testosterone or DHEAS levels (Model 2 and 3); however, there was a trend towards significance between values of DHEAS and FMD (b-coefficient, −0.160, 95% CI: −0.340 to −0.045, *p*-value = 0.053). Model 4 showed that FMD was associated with levels of insulin (b-coefficient = −0.166, 95% CI: −0.322 to -0.093, *p*-value = 0.034), testosterone (b-coefficient = −0.165, 95% CI: −0.395 to 0.038, *p*-value = 0.034), DHEAS (b-coefficient = −0.187, 95% CI: −0.362 to −0.093, *p*-value = 0.017). Further adjustment for FSH in any of the beforementioned models did not substantially change the results. The results remained the same, restricting the analysis in women with FMD values within the laboratory-reference range.

**TABLE 3:** Linear regression analysis included multivariable models fitted with the most significant predictors of flow-mediated dilation.

	Model R2 (%)	b-coefficient	95% CI	*p*-value
**Model 1**				
Age (years)	5.4	−0.073	−0.238 to −0.064	0.449
Pulse pressure (mmHg)		−0.011	−0.321 to 0.038	0.924
Smoking		0.081	0.056 to 0.278	0.401
LDL-C (mg/dl)		−0.001	−0.412 to 0.089	0.993
Insulin (μIU/ml)		−0.172	−0.368 to −0.063	0.085
BMI (kg/m^2^)		−0.026	−0.378 to 0.032	0.789
HSP60		**0.171**	**0.043 to 0.358**	**0.046**
**Model 2**				
Age (years)	2.5	−0.062	−0.268 to 0.037	0.438
Pulse pressure (mmHg)		−0.047	−0.142 to 0.094	0.551
Smoking		−0.029	−0.142 to 0.328	0.715
LDL-C (mg/dl)		−0.040	−0.418 to 0.275	0.611
Insulin (μIU/ml)		−0.146	−0.357 to −0.006	0.064
BMI (kg/m2)		0.005	−0.042 to 0.184	0.953
HSP60		0.128	−0.052 to 0.421	0.105
Testosterone (ng/dl)		−**0.158**	−**0.305 to 0.099**	**0.047**
**Model 3**				
Age (years)	3.3	−0.052	−0.326 to 0.048	0.535
Pulse pressure (mmHg)		−0.029	−0.328 to 0.121	0.731
Smoking		0.019	−0.094 to 0.211	0.817
LDL-C (mg/dl)		−0.036	−0.279 to 0.189	0.668
Insulin (μIU/ml)		−0.132	−0.367 to 0.083	0.113
BMI (kg/m^2^)		−0.014	−0.184 to 0.327	0.869
HSP60		**0.181**	**0.048 to 0.466**	**0.030**
DHEAS (ng/dl)		−0.160	−0.354 to −0.045	0.053
**Model 4**				
Age (years)	8.0	−0.049	−0.258 to 0.009	0.535
Pulse pressure (mmHg)		−0.029	−0.198 to 0.205	0.711
Smoking		−0.025	−0.274 to 0.178	0.753
LDL-C (mg/dl)		0.008	−0.047 to 0.149	0.921
Insulin (μIU/ml)		−**0.166**	−**0.322 to** −**0.093**	**0.034**
BMI (kg/m^2^)		0.096	0.003 to 0.389	0.271
HSP60		0.141	−0.093 to 0.338	0.066
Testosterone		−**0.165**	−0.395 to 0.038	**0.034**
DHEAS (ng/dl)		−**0.187**	−**0.362 to** −**0.093**	**0.017**

*Adjustment for values of follicle-stimulating hormone did not substantially change the results of the above models. Bold indicates statistical significance, which was defined as *p*-value < 0.05.

SBP, systolic blood pressure; LDL-C, low density lipoprotein cholesterol; BMI, body mass index; HSP60, heat shock protein 60; DHEAS, dehydroepiandrosterone; Model 2, Model 1 + testosterone; Model 3, Model 1 + DHEAS; Model 4, Model 3 + DHEAS.

## Discussion

The results of this study support a significant positive association between FMD and HSP60 values after adjustment for cardiovascular risk factors. However, this association became non-significant after further adjustment for androgens and insulin levels, indicating a possible correlation between testosterone and HSP60. Moreover, FSH levels seem to be directly associated with levels of HSP60 after the menopausal transition, independently of cardiovascular risk factors.

Heat shock proteins (HSP) have a predominant role in modifying the cellular response to stress-related events (Van Hinsbergh and Koolwijk, 2008). HSP60 molecules are evident intracellular (e.g., mitochondria matrix space and cytoplasm) and in the plasma membrane and extracellular space, and the blood circulation (Duan et al., 2020). Consequently, the functional role of HSP60 molecules is related to their localization. It varies from chaperoning mitochondrial activity to modification of cellular processes like immune responses, cell proliferation and cell apoptosis (Henderson et al., 2013).

Recent data suggest a complex role of HSP60, which belong to the chaperonins of Group I and typically functions inside the mitochondria. The role of this molecule ranges from a proinflammatory biomarker of vascular damage up to an anti-inflammatory immunomodulator (Caruso Bavisotto et al., 2020; Krishnan-Sivadoss et al., 2021). The role of HSP60 in the survival of endothelial cells *in vivo*, especially in the context of the hormonal milieu following the menopausal transition, has received limited attention. The expression and localization of HSP60 in vascular endothelial cells are regulated by various insults like inflammation, chemical stress and infectious agents (Caruso Bavisotto et al., 2020; Duan et al., 2020). Results are mainly available from *in vitro* rather than *in vivo* studies, highlighting that risk factors for atherogenesis may upregulate the intracellular expression of HSP60 and induce translocation of this protein from the mitochondria to the cell surface. The level of intracellular and especially extracellular HSP60 seems to be mediating the additional atherogenic risk. HSP60 molecules bind to the membrane ATP-synthase, serving a protective role against cell apoptosis (Duan et al., 2020). Overexpression of intra- and extracellular HSP60 has been reported to promote the production of proteolytic cytokines and enzymes, like TNF and adhesion molecules, which possibly induce the proliferation and migration of vascular smooth muscle cells leading to atherogenesis (Duan et al., 2020).

In our sample, serum levels of androgenic sex hormones were not significantly associated with HSP60, but women with higher FSH values did have higher HSP60 levels, irrespectively to traditional cardiovascular risk factors. This interesting association implies that the FSH increase evident around the time of the menopausal transition and even before ovarian senescence (Harlow et al., 2012) is linked with changes in HSP-homeostasis. This finding is in line with previous *in vitro* evidence describing maximum HSP60 production after the ovulation, immediately following the peak of FSH values observed within the menstrual cycle (Neuer et al., 2000). In line with this observation, a small interventional study of 90 postmenopausal women reported a significant reduction in titers of antibodies against HSP60 in women receiving treatment with hormone replacement as opposed to control (Rajtar-Ciosek et al., 2015) suggesting a hormonal regulation of HSP-levels.

We observed a positive association between FMD levels and serum HSP60 antigen levels in postmenopausal women with FMD within the reference range. This interaction, however, became non-significant after further adjustment for insulin resistance and androgen levels. Earlier research has shown that HSP60s are involved in mitochondrial steroidogenesis by regulating cholesterol transport in human placenta cells (Miller, 2013). In this context, the hypothesis of the possible mediating effect of serum androgens on the interaction between HSP60 and FMD seems not irrational. An earlier study (Wick, 2016) describes the hypothesis of an atheroprotective immune tolerance, which may be induced by activating cellular and humoral immune reactions to HSP60 autoantigens. According to our findings, insulin resistance, defined as HOMA-IR, and levels of androgens mediated the association between HSP60 and FMD in the multivariable analysis. The postmenopausal profile of “relative androgenicity” following ovarian senescence is likely to act as the trigger to promote athero-protective immune tolerance to HSP60 molecules (Davis et al., 2015). Data on the link between HSP60 production in vascular endothelial cells and androgens remain conflicting. Levels of HSP60 autoantigen seem to be modulated according to serum levels of circulating androgens, as reported in a study of human prostate cells, where autoantigen levels have been reported to increase by 1.7-fold in androgen-deplete cells but not in androgen-sensitive cells (Wright et al., 2003). On the other hand, *in vitro* data in Sertoli cells, retrieved from mice, demonstrated evidence of testosterone-induced down-regulation of HSP60, mediated by the inhibition of the transcription of the heat shock factor-1 (Yang et al., 2014).

This study has certain limitations that should be outlined. First, the sample size is small. Second, the cross-sectional nature of the observed associations cannot permit causality detection. Third, we have not measured HSP60 antibody levels, which would provide more insight into the possible interaction between this chaperonin molecule and endothelial function. Fourth, data on HSP60 levels of premenopausal women were not available. However, in a previous study (Kim et al., 2009) report that premenopausal women have lower HSP60 levels in comparison to postmenopausal women. On the other side, our study group consists of carefully selected postmenopausal women with no apparent health problems, enabling us to minimize the risk of bias in the observed investigations.

In conclusion, we observed that endothelial function in postmenopausal women is associated positively with HSP60 levels, indicating a possible association irrespectively of traditional cardiovascular risk factors or insulin. As expected, endothelial function was negatively associated with testosterone and insulin. The combined assessment of the effect of HSP60 levels in addition to other hormonal factors like testosterone, insulin and DHEAS into the equation results in losing this balance between HSP60 and FMD, and subsequent vascular damage. These observations are implying a possible association between testosterone and HSP60 levels in postmenopausal women. Further prospective studies are needed to confirm the significance of our findings. If the results of this study were proven to be causal, this would possibly reveal a new pathophysiological mechanism explaining the effect of androgens on endothelial cells in this group of women with non-elevated cardiovascular risk.

## Data Availability

The raw data supporting the conclusions of this article will be made available by the authors, without undue reservation.

## References

[B1] Cappello F. Marino Gammazza A. Palumbo Piccionello A. Campanella C. Pace A. Conway de Macario E. (2014). Hsp60 Chaperonopathies and Chaperonotherapy: Targets and Agents. Expert Opin. Ther. Targets 18, 185–208. 10.1517/14728222.2014.856417 24286280

[B2] Caruso Bavisotto C. Alberti G. Vitale A. M. Paladino L. Campanella C. Rappa F. (2020). Hsp60 Post-Translational Modifications: Functional and Pathological Consequences. Front. Mol. Biosci. 7. 10.3389/fmolb.2020.00095 PMC7289027 32582761

[B3] Coelho V. Faria A. M. C. (2012). HSP60: Issues and Insights on its Therapeutic Use as an Immunoregulatory Agent. Front. Immun. 2, 97. 10.3389/fimmu.2011.00097 PMC3342027 22566886

[B4] Creatsa M. Armeni E. Stamatelopoulos K. Rizos D. Georgiopoulos G. Kazani M. (2012). Circulating Androgen Levels Are Associated with Subclinical Atherosclerosis and Arterial Stiffness in Healthy Recently Menopausal Women. Metabolism 61, 193–201. 10.1016/j.metabol.2011.06.005 21820132

[B5] Davis S. R. Lambrinoudaki I. Lumsden M. Mishra G. D. Pal L. Rees M. (2015). Menopause. Nat. Rev. Dis. Prim. 1. 10.1038/nrdp.2015.4 27188659

[B6] Deniset J. F. Hedley T. E. Hlaváčková M. Chahine M. N. Dibrov E. O'Hara K. (2018). Heat Shock Protein 60 Involvement in Vascular Smooth Muscle Cell Proliferation. Cell. Signal. 47, 44–51. 10.1016/j.cellsig.2018.03.011 29596871

[B7] Duan Y. Tang H. Mitchell-silbaugh K. Fang X. Han Z. Ouyang K. (2020). Heat Shock Protein 60 in Cardiovascular Physiology and Diseases. Front. Mol. Biosci. 7. 10.3389/fmolb.2020.00073 PMC7203681 32426370

[B8] Garrido C. Gurbuxani S. Ravagnan L. Kroemer G. (2001). Heat Shock Proteins: Endogenous Modulators of Apoptotic Cell Death. Biochem. Biophysical Res. Commun. 286, 433–442. 10.1006/bbrc.2001.5427 11511077

[B9] Georgiopoulos G. A. Lambrinoudaki I. Athanasouli F. Armeni E. Rizos D. Kazani M. (2016). Free Androgen Index as a Predictor of Blood Pressure Progression and Accelerated Vascular Aging in Menopause. Atherosclerosis 247, 177–183. 10.1016/j.atherosclerosis.2016.02.021 26922717

[B10] Harlow S. D. Gass M. Hall J. E. Lobo R. Maki P. Rebar R. W. (2012). Executive Summary of the Stages of Reproductive Aging Workshop + 10: Addressing the Unfinished Agenda of Staging Reproductive Aging. Fertil. Steril. 97, 843–851. 10.1016/j.fertnstert.2012.01.128 22341880 PMC3340904

[B11] Henderson B. Fares M. A. Lund P. A. (2013). Chaperonin 60: A Paradoxical, Evolutionarily Conserved Protein Family with Multiple Moonlighting Functions. Biol. Rev. 88, 955–987. 10.1111/brv.12037 23551966

[B12] Hollander J. M. Lin K. M. Scott B. T. Dillmann W. H. (2003). Overexpression of PHGPx and HSP60/10 Protects Against Ischemia/Reoxygenation Injury. Free Radic. Biol. Med. 35, 742–751. 10.1016/s0891-5849(03)00400-3 14583338

[B13] Inaba Y. Chen J. A. Bergmann S. R. (2010). Prediction of Future Cardiovascular Outcomes by Flow-Mediated Vasodilatation of Brachial Artery: A Meta-Analysis. Int. J. Cardiovasc. Imaging 26, 631–640. 10.1007/s10554-010-9616-1 20339920

[B34] Kim Y. S. Koh J.-M. Lee Y.-S. Kim B.-J. Lee S. H. Lee K.-U. (2009). Increased Circulating Heat Shock Protein 60 Induced by Menopause, Stimulates Apoptosis of Osteoblast Lineage Cells via Up-Regulation of Toll-Like Receptors. Bone 45, 68–76. 10.1016/j.bone.2009.03.658 19306954

[B14] Krishnan‐Sivadoss I. Mijares‐Rojas I. A. Villarreal‐Leal R. A. Torre‐Amione G. Knowlton A. A. Guerrero‐Beltrán C. E. (2021). Heat Shock Protein 60 and Cardiovascular Diseases: An Intricate Love‐Hate Story. Med. Res. Rev. 41, 29–71. 10.1002/med.21723 32808366 PMC9290735

[B15] Miller W. L. (2013). Steroid Hormone Synthesis in Mitochondria. Mol. Cell. Endocrinol. 379, 62–73. 10.1016/j.mce.2013.04.014 23628605

[B16] Moghimpour Bijani F. Vallejo J. G. Rezaei N. (2012). Toll-Like Receptor Signaling Pathways in Cardiovascular Diseases: Challenges and Opportunities. Int. Rev. Immunol. 31, 379–395. 10.3109/08830185.2012.706761 23083347

[B17] Monreal-Flores J. Espinosa-García M. T. García-Regalado A. Arechavaleta-Velasco F. Martínez F. (2017). The Heat Shock Protein 60 Promotes Progesterone Synthesis in Mitochondria of JEG-3 Cells. Reprod. Biol. 17, 154–161. 10.1016/j.repbio.2017.04.001 28434777

[B18] Moreau K. L. Hildreth K. L. Meditz A. L. Deane K. D. Kohrt W. M. (2012). Endothelial Function Is Impaired Across the Stages of the Menopause Transition in Healthy Women. J. Clin. Endocrinol. Metab. 97, 4692–4700. 10.1210/jc.2012-2244 22969140 PMC3513538

[B19] Neuer A. Lam K. N. Tiller F. W. Kiesel L. Witkin S. S. (1997). Humoral Immune Response to Membrane Components of *Chlamydia trachomatis* and Expression of Human 60 kDa Heat Shock Protein in Follicular Fluid of *In-Vitro* Fertilization Patients. Hum. Reprod. 12, 925–929. 10.1093/humrep/12.5.925 9194641

[B20] Neuer A. Spandorfer S. D. Giraldo P. Dieterle S. Rosenwaks Z. Witkin S. S. (2000). The Role of Heat Shock Proteins in Reproduction. Hum. Reprod. Update 6, 149–159. 10.1093/humupd/6.2.149 10782573

[B21] Olvera-Sanchez S. Espinosa-Garcia M. T. Monreal J. Flores-Herrera O. Martinez F. (2011). Mitochondrial Heat Shock Protein Participates in Placental Steroidogenesis. Placenta 32, 222–229. 10.1016/j.placenta.2010.12.018 21232789

[B22] Rajtar-Ciosek A. Kacalska-Janssen O. Zmaczyński A. Wyroba J. Tomczyk R. Wiatr J. (2015). Reduction in the Level of Antibodies Against Heat Shock Proteins 60 During Different Hormonal Protocols in Postmenopausal Women. pm 4, 218–222. 10.5114/pm.2015.56402 PMC4733899 26848292

[B23] Rockwood K. Song X. MacKnight C. Bergman H. Hogan D. B. McDowell I. (2005). A Global Clinical Measure of Fitness and Frailty in Elderly People. Can. Med. Assoc. J. 173, 489–495. 10.1503/cmaj.050051 16129869 PMC1188185

[B24] Stamatelopoulos K. Georgiopoulos G. Athanasouli F. Nikolaou P.-E. Lykka M. Roussou M. (2019). Reactive Vasodilation Predicts Mortality in Primary Systemic Light-Chain Amyloidosis. Circ. Res. 125, 744–758. 10.1161/CIRCRESAHA.119.314862 31401949 PMC6784773

[B25] Stamatelopoulos K. S. Georgiopoulos G. A. Sfikakis P. P. Kollias G. Manios E. Mantzou E. (2011). Pilot Study of Circulating Prolactin Levels and Endothelial Function in Men with Hypertension. Am. J. Hypertens. 24, 569–573. 10.1038/ajh.2011.16 21331059

[B26] Stamatelopoulos K. Sibbing D. Rallidis L. S. Georgiopoulos G. Stakos D. Braun S. (2015). Amyloid-Beta (1-40) and the Risk of Death from Cardiovascular Causes in Patients with Coronary Heart Disease. J. Am. Coll. Cardiol. 65, 904–916. 10.1016/j.jacc.2014.12.035 25744007

[B27] Thijssen D. H. J. Bruno R. M. van Mil A. C. C. M. Holder S. M. Faita F. Greyling A. (2019). Expert Consensus and Evidence-Based Recommendations for the Assessment of Flow-Mediated Dilation in Humans. Eur. Heart J. 40, 2534–2547. 10.1093/eurheartj/ehz350 31211361

[B28] Van Hinsbergh V. W. M. Koolwijk P. (2008). Endothelial Sprouting and Angiogenesis: Matrix Metalloproteinases in the Lead. Cardiovasc. Res. 78, 203–212. 10.1093/cvr/cvm102 18079100

[B29] Wick C. (2016). Tolerization against Atherosclerosis Using Heat Shock Protein 60. Cell Stress Chaperones 21, 201–211. 10.1007/s12192-015-0659-z 26577462 PMC4786533

[B30] Wright M. E. Eng J. Sherman J. Hockenbery D. M. Nelson P. S. Galitski T. (2003). Identification of Androgen-Coregulated Protein Networks from the Microsomes of Human Prostate Cancer Cells. Genome Biol. 5, R4. 10.1186/gb-2003-5-1-r4 14709176 PMC395736

[B31] Xu Y. Arora R. C. Hiebert B. M. Lerner B. Szwajcer A. McDonald K. (2014). Non-Invasive Endothelial Function Testing and the Risk of Adverse Outcomes: A Systematic Review and Meta-Analysis. Eur. Hear. J. Cardiovasc. Imaging 15, 736–746. 10.1093/ehjci/jet256 24399339

[B32] Yang L. Wang Y. Zhang Q. Lai Y. Li C. Zhang Q. (2014). Identification of *Hsf1* as a Novel Androgen Receptor-Regulated Gene in Mouse Sertoli Cells. Mol. Reprod. Dev. 81, 514–523. 10.1002/mrd.22318 24599545

[B33] Zhao Y. Zhang C. Wei X. Li P. Cui Y. Qin Y. (2015). Heat Shock Protein 60 Stimulates the Migration of Vascular Smooth Muscle Cells via Toll-Like Receptor 4 and ERK MAPK Activation. Sci. Rep. 5, 15352. 10.1038/srep15352 26477505 PMC4609986

